# Supplementary material for: Global distribution, trends, and drivers of flash drought occurrence
Source: Nat Commun. 2021 Nov 3;12:6330. doi: 10.1038/s41467-021-26692-z (PMC8566603; doi:10.1038/s41467-021-26692-z)
Supplement: Supplementary file 1 — Supplementary Information [file 41467_2021_26692_MOESM1_ESM.pdf]

Supplementary Information for

Global Distribution, Trends, and Drivers of Flash Drought Occurrence

Jordan I. Christian[a,*], Jeffrey B. Basara[a,b], Eric D. Hunt[c], Jason A. Otkin[d], Jason C. Furtado[a], Vimal Mishra[e,f], Xiangming Xiao[g], Robb M. Randall[h]

[a]School of Meteorology, University of Oklahoma, Norman, Oklahoma, United States

[b]School of Civil Engineering and Environmental Science, University of Oklahoma, Norman, Oklahoma, United States

[c]Atmospheric and Environmental Research, Inc., Lexington, Massachusetts

[d]Cooperative Institute for Meteorological Satellite Studies, Space Science and Engineering Center, University of Wisconsin-Madison, Madison, Wisconsin

[e]Civil Engineering, Indian Institute of Technology (IIT), Gandhinagar, India

[f]Earth Sciences, Indian Institute of Technology (IIT), Gandhinagar, India

[g]Department of Microbiology and Plant Biology, Center of Spatial Analysis, University of Oklahoma, Norman, OK

[h]CCDC Army Research Laboratory, White Sands Missile Range, New Mexico

Corresponding author: Jordan Christian

Address: School of Meteorology, University of Oklahoma, Norman, OK

Phone number: 405-325-6561

Email address: jchristian@ou.edu

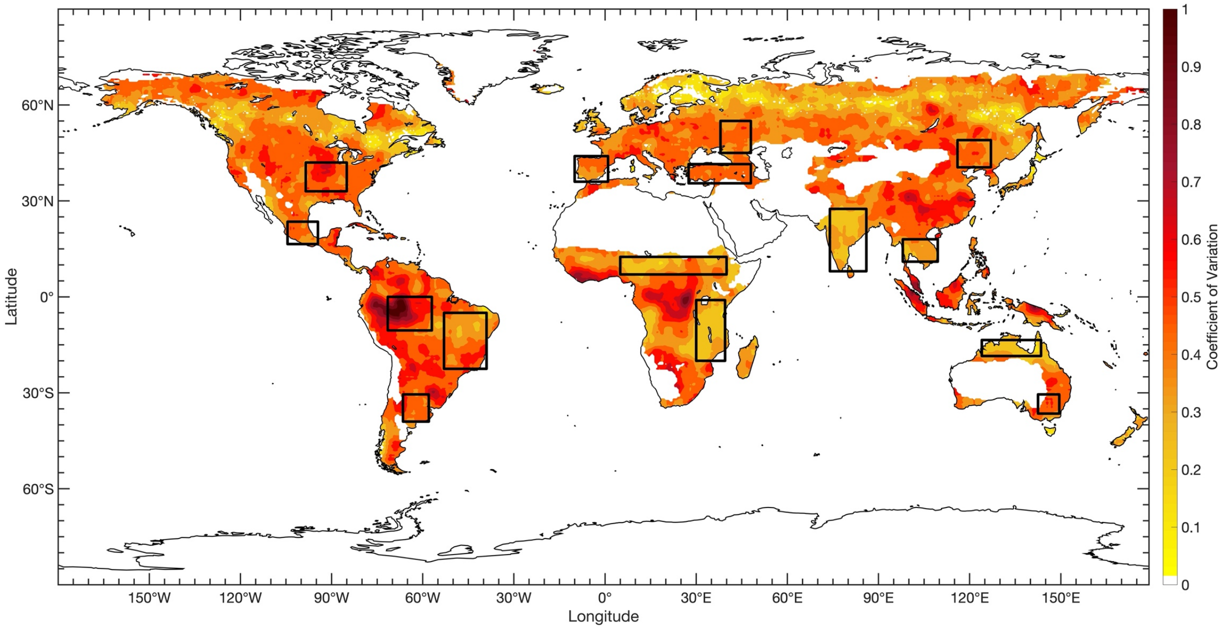

Supplementary Figure 1. Variability of flash drought occurrence between datasets. Coefficient of variation for the percent of years with a flash drought (1980-2015) between MERRA, MERRA-2, ERA-Interim, and ERA5.

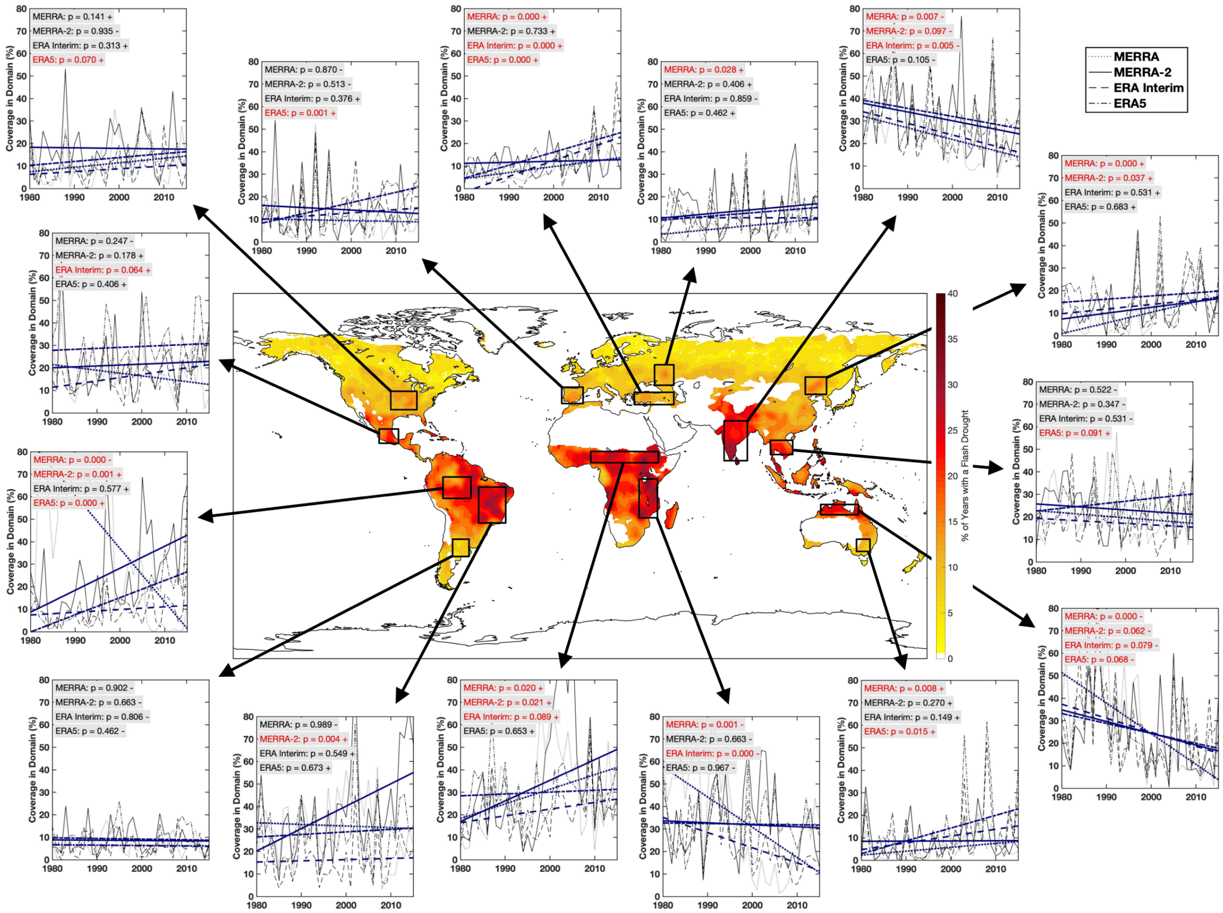

Supplementary Figure 2. Trends in flash drought occurrence for each dataset. Flash drought spatial coverage (percent) for each of the domains outlined in black on the map. Each black line in the time series plot represents flash drought spatial coverage from each of the four reanalyses, while the thicker blue lines represent the trend line for each reanalysis. P-values highlighted in red are those that are statistically significant at the 90% confidence level using the Mann-Kendall test.

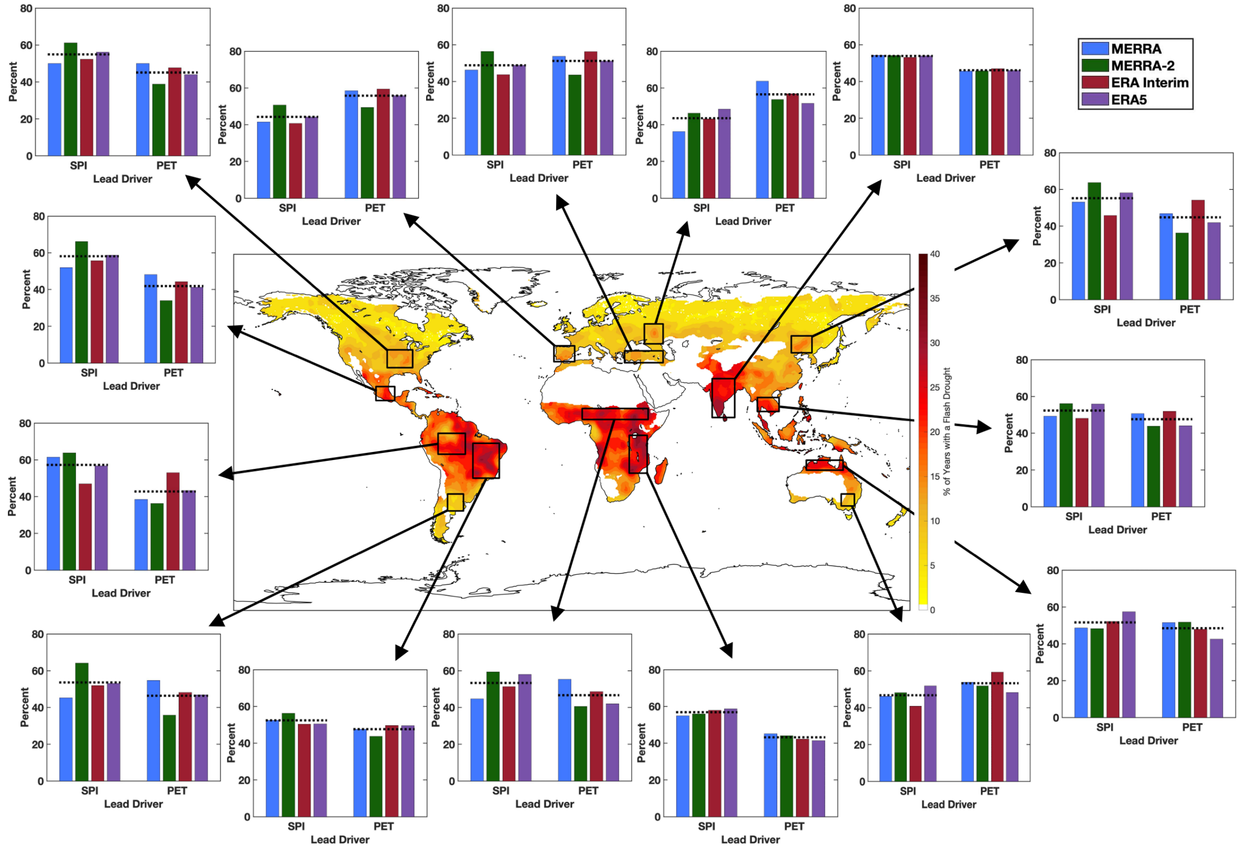

Supplementary Figure 3. Lead driver of flash drought occurrence. Percentage of flash drought events with SPI or PET as the lead driver during flash drought development from the four reanalysis datasets (different colored bars) for each of the domains outlined in black on the map. The black dotted lines represent the mean between all four reanalysis datasets.

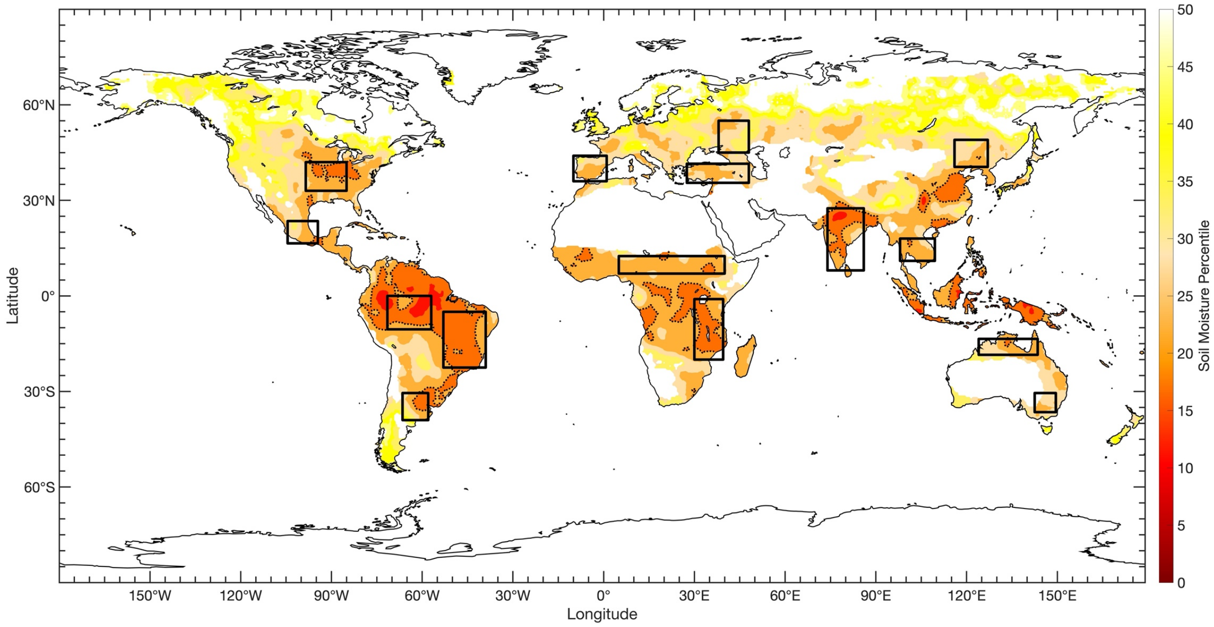

Supplementary Figure 4. Soil moisture conditions at the end of flash drought. Mean soil moisture percentile at the end of flash droughts between 1980 and 2015 from the four reanalysis datasets. The dotted black line represents the contour for the 20th percentile.

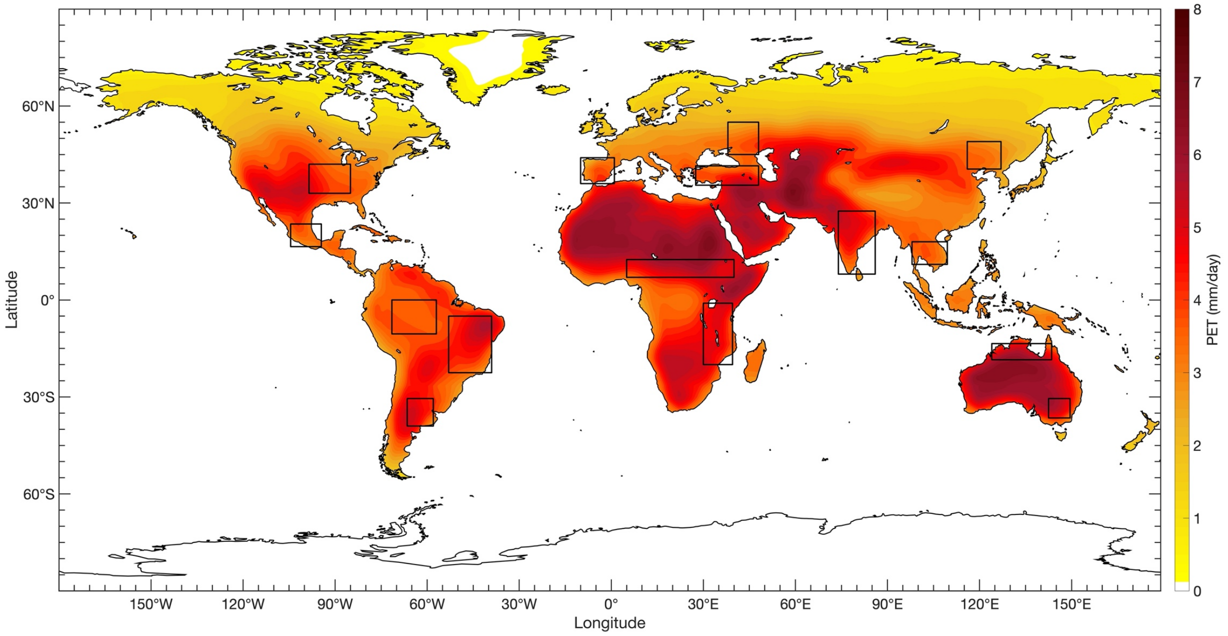

Supplementary Figure 5. Daily average PET. Daily average PET during the growing season for latitudes greater than 30° from the equator (March through October in Northern Hemisphere and September through April in Southern Hemisphere) and year-round for latitudes in the tropics and subtropics (between 30°S and 30°N) from MERRA-2 between 1980 and 2015.

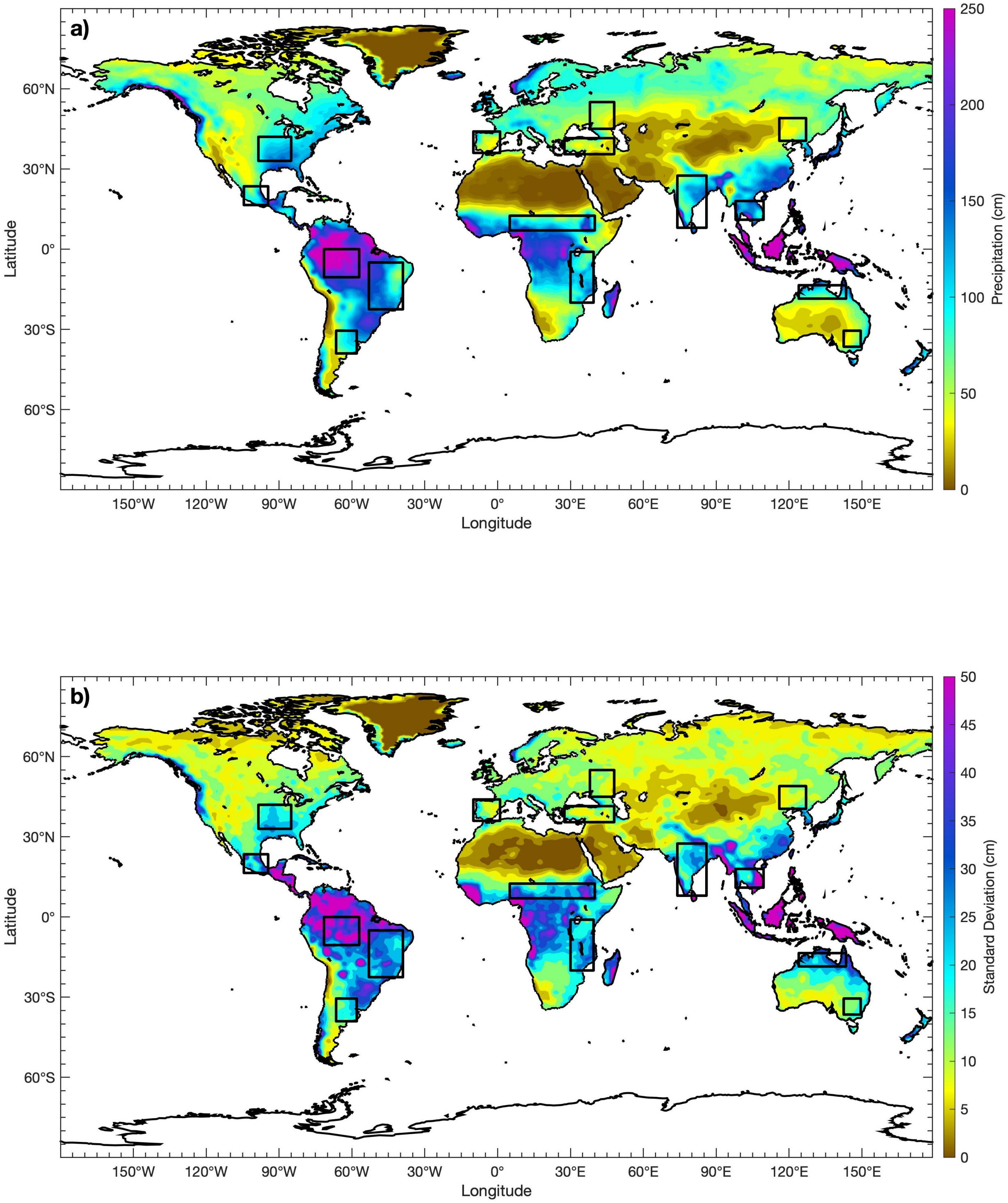

Supplementary Figure 6. Magnitude and variability of precipitation. (a) Average annual precipitation and (b) interannual variability of precipitation from MERRA-2 between 1980 and 2015.

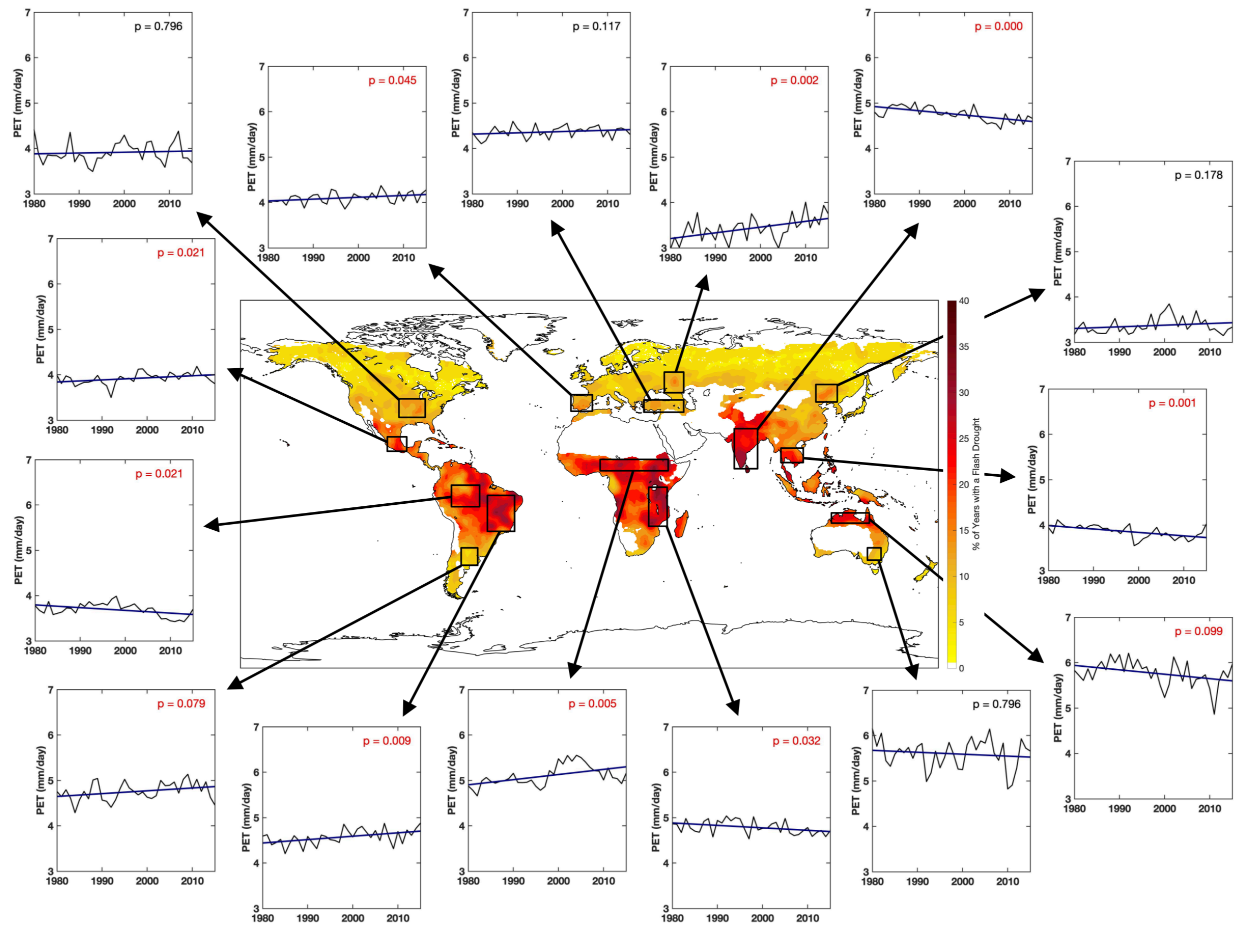

Supplementary Figure 7. Trends in PET. Daily average PET (mm/day; black line) during the growing season for domains in latitudes greater than 30° from the equator (March through October in Northern Hemisphere and September through April in Southern Hemisphere) and year-round for domains in latitudes in the tropics and subtropics (between 30°S and 30°N) from MERRA-2. The domains are outlined in black on the map. The thicker blue line represents the trend line for PET. P-values highlighted in red are statistically significant trends at the 90% confidence level using the Mann-Kendall test.

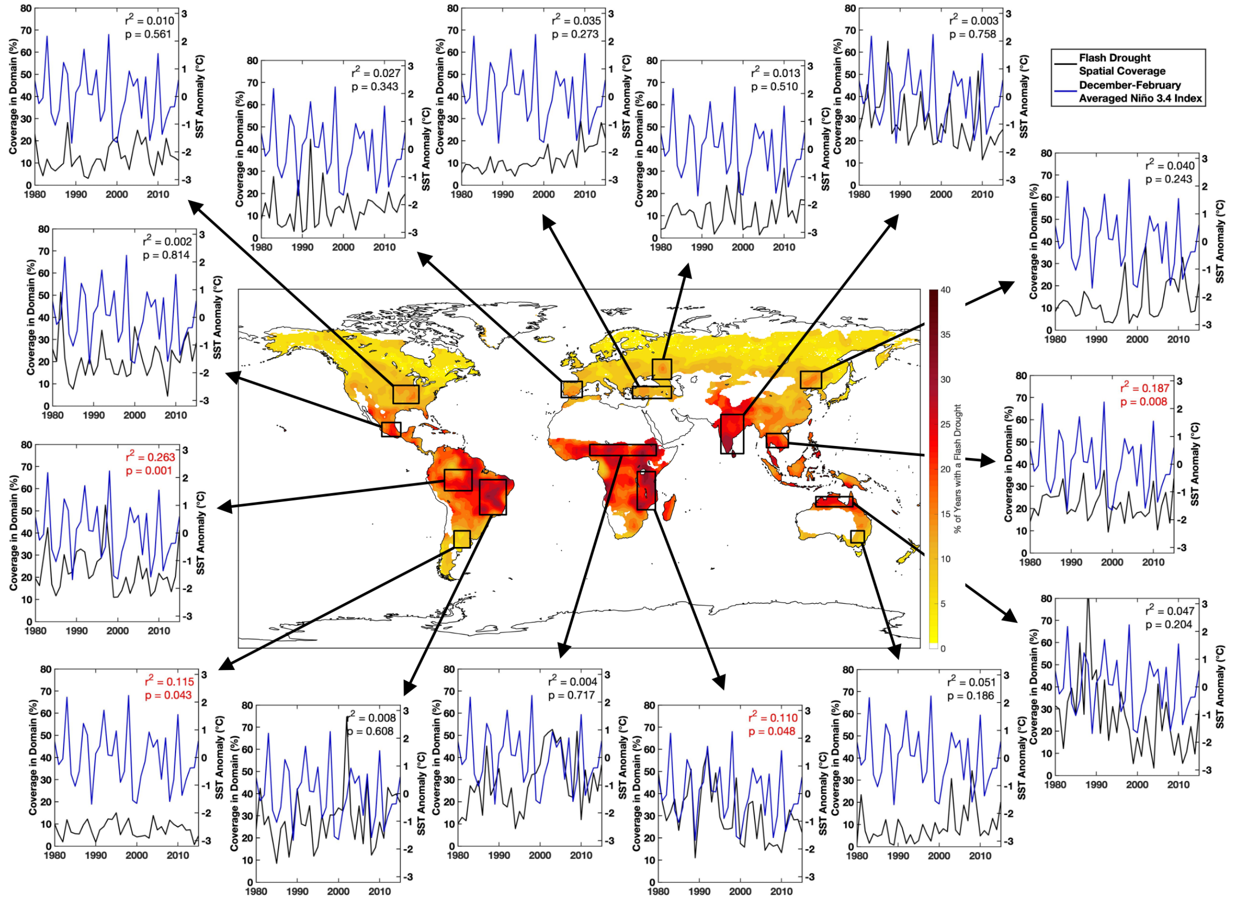

Supplementary Figure 8. Relationship between flash drought occurrence and ENSO. Mean flash drought spatial coverage (percent) from the four reanalysis datasets (black line) for each of the domains outlined in black on the map and the December – February averaged Niño 3.4 index (blue line). P-values highlighted in red indicate a statistically significant correlation between flash drought spatial coverage and SST anomalies at the 90% confidence level.

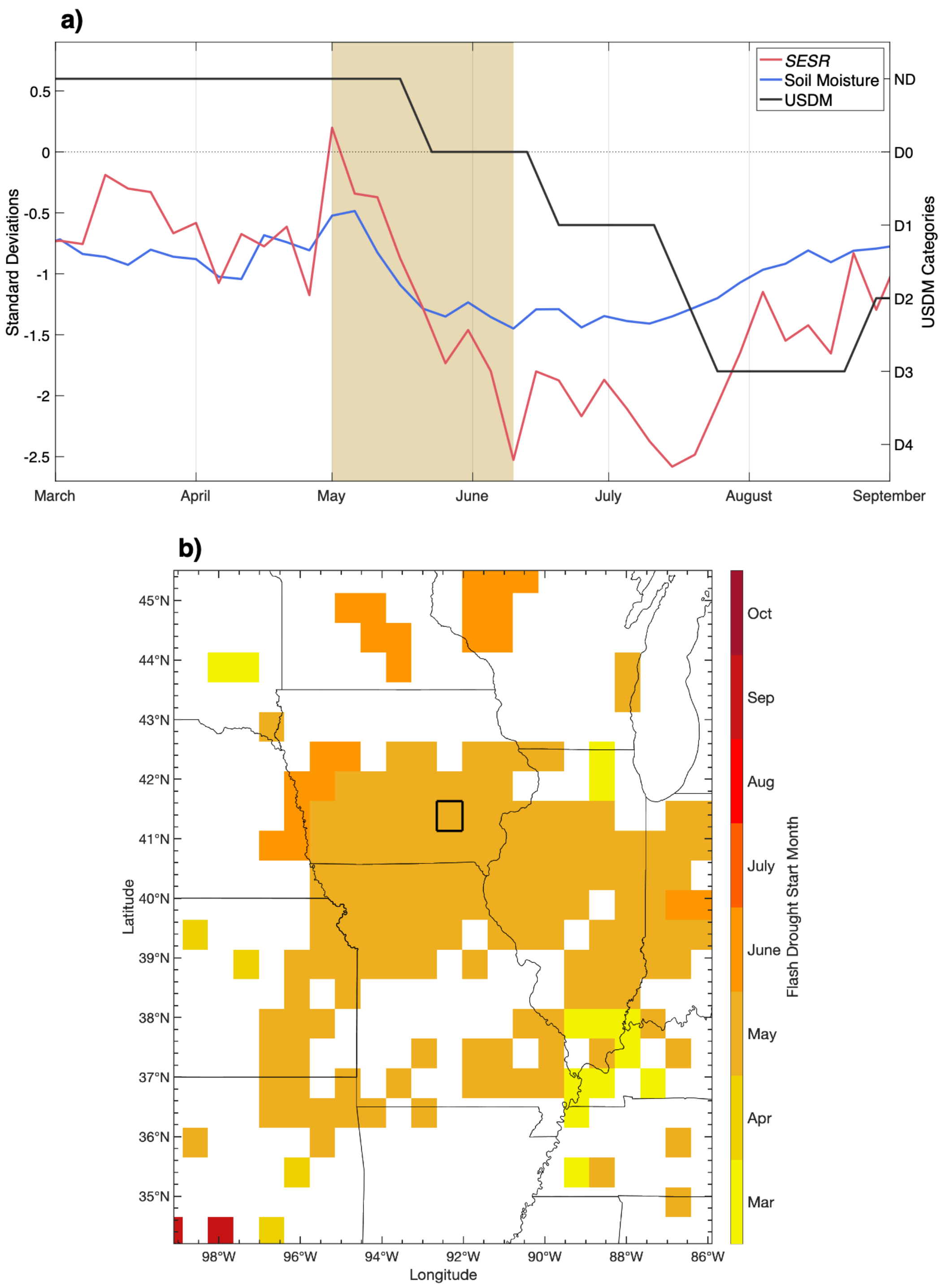

Supplementary Figure 9. Temporal and spatial evolution of the 2012 flash drought in the central United States. (a) The time series shows *SESR* and 0-100 cm standardized soil moisture from MERRA-2, as well as the USDM drought category in southeastern Iowa, United States during 2012. The tan color indicates the time period of flash drought in (a). (b) The spatial plot shows the month in which flash drought began. The black outline in (b) shows the location of the grid point used in (a).

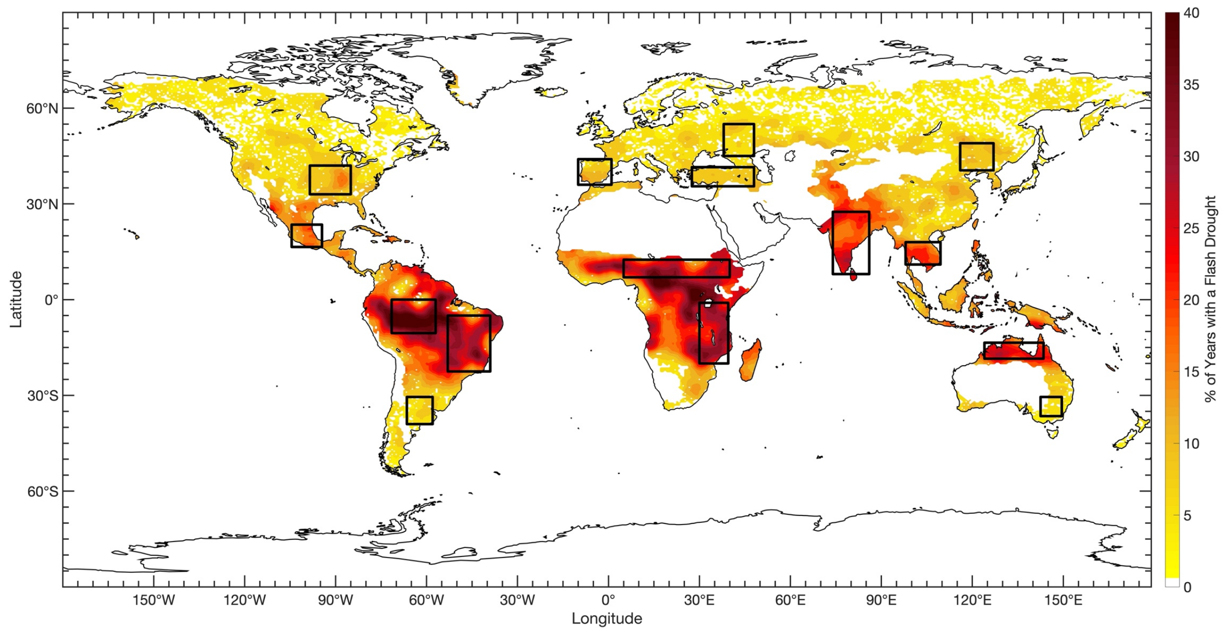

Supplementary Figure 10. Flash drought occurrence from MERRA. Percent of years with a flash drought between 1980 and 2015 for the MERRA dataset.

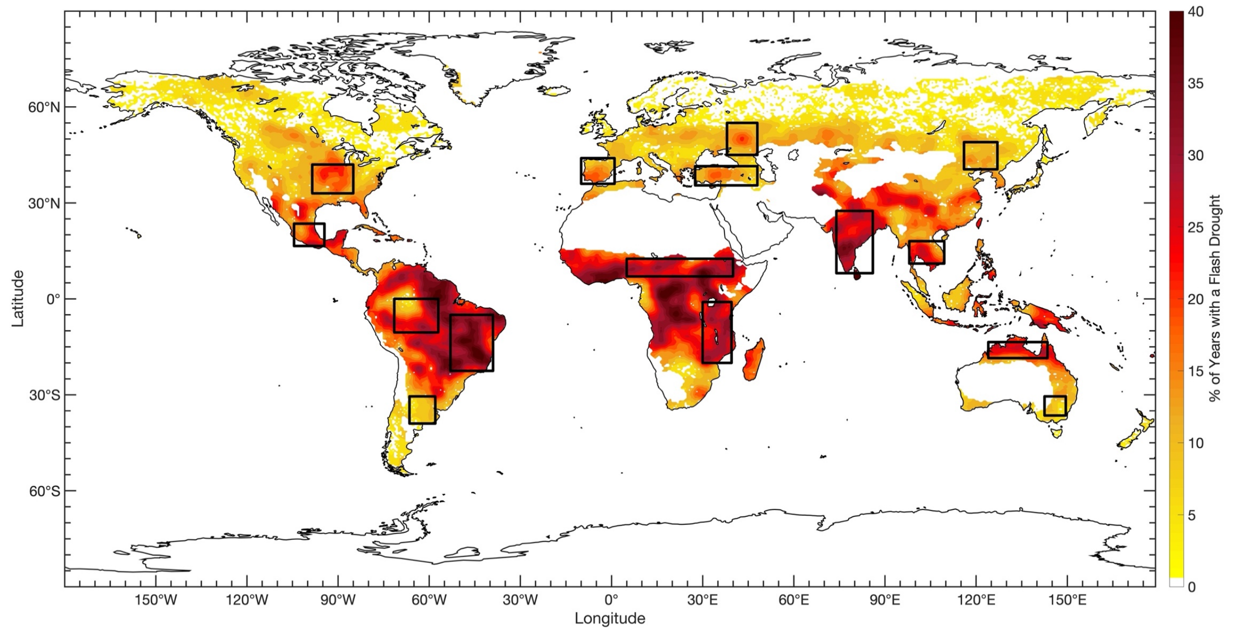

Supplementary Figure 11. Flash drought occurrence from MERRA-2. The same as Supplementary Figure 10, but for the MERRA-2 dataset.

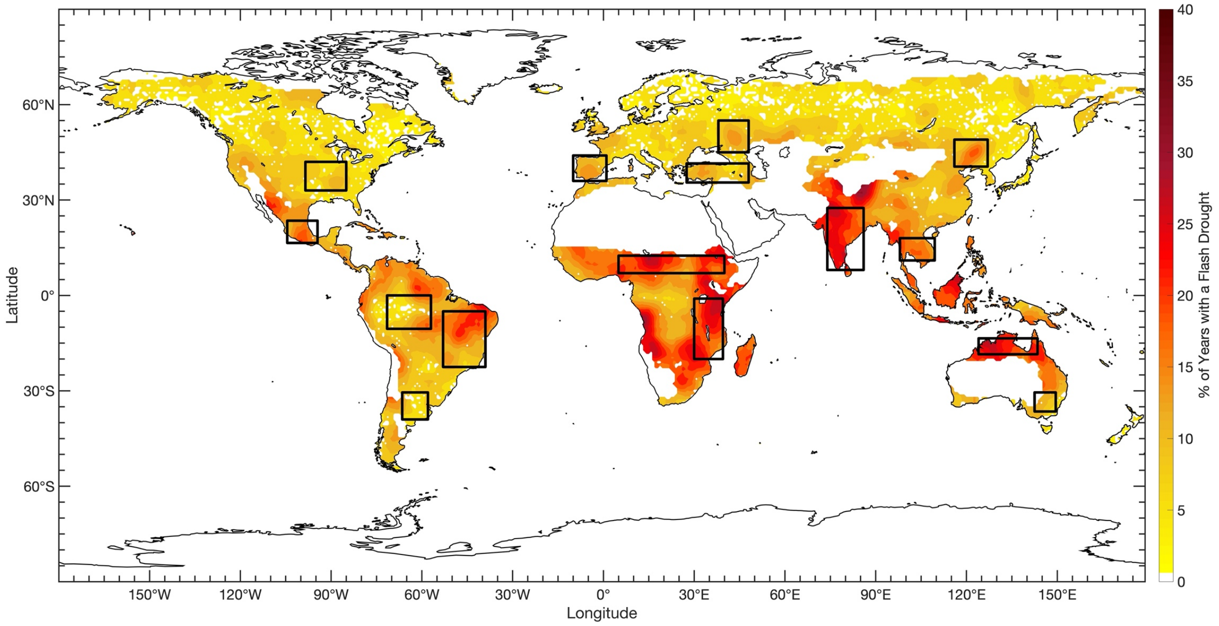

Supplementary Figure 12. Flash drought occurrence from ERA-Interim. The same as Supplementary Figure 10, but for the ERA-Interim dataset.

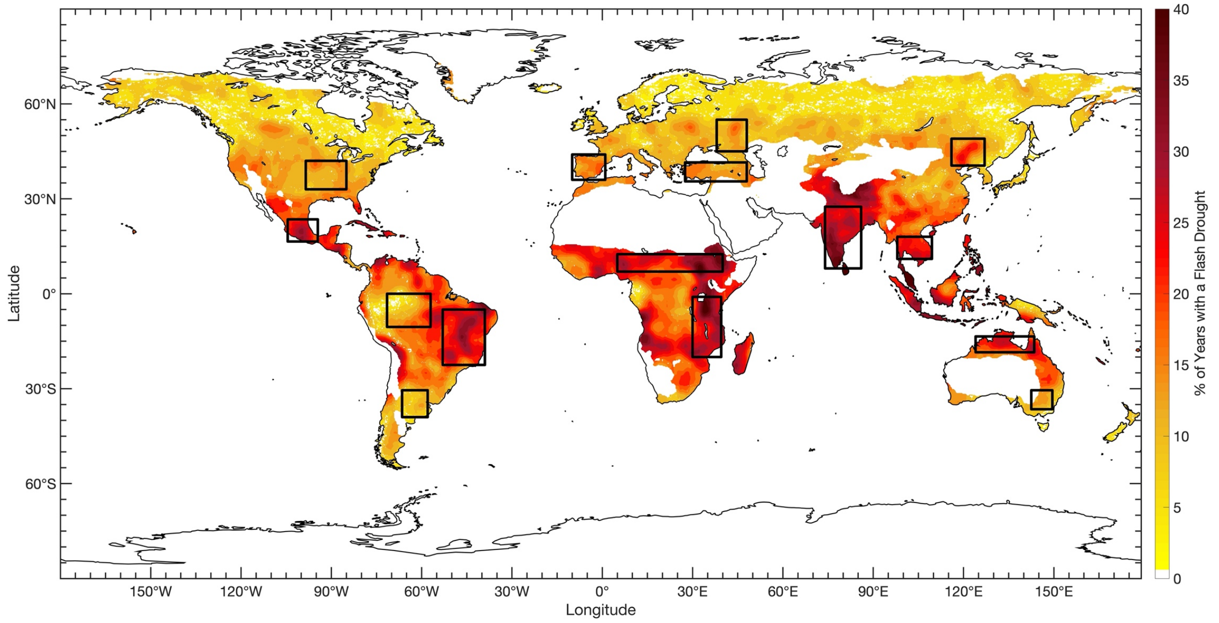

Supplementary Figure 13. Flash drought occurrence from ERA5. The same as Supplementary Figure 10, but for the ERA5 dataset.
